# Susceptibility and Resistance Mechanisms During Photodynamic Therapy of Melanoma

**DOI:** 10.3389/fonc.2020.00597

**Published:** 2020-05-12

**Authors:** Xin-Ying Li, Liu-Chang Tan, Li-Wen Dong, Wan-Qi Zhang, Xiao-Xiao Shen, Xiao Lu, Hong Zheng, Yuan-Gang Lu

**Affiliations:** ^1^Department of Plastic Surgery, Daping Hospital, Army Medical University, Chongqing, China; ^2^Department of Thoracic Surgery, Xinqiao Hospital, Army Medical University, Chongqing, China

**Keywords:** photodynamic therapy, melanoma, apoptosis, autophagy, tumor immunity

## Abstract

Melanoma is the most aggressive malignant skin tumor and arises from melanocytes. The resistance of melanoma cells to various treatments results in rapid tumor growth and high mortality. As a local therapeutic modality, photodynamic therapy has been successfully applied for clinical treatment of skin diseases. Photodynamic therapy is a relatively new treatment method for various types of malignant tumors in humans and, compared to conventional treatment methods, has fewer side effects, and is more accurate and non-invasive. Although several *in vivo* and *in vitro* studies have shown encouraging results regarding the potential benefits of photodynamic therapy as an adjuvant treatment for melanoma, its clinical application remains limited owing to its relative inefficiency. This review article discusses the use of photodynamic therapy in melanoma treatment as well as the latest progress made in deciphering the mechanism of tolerance. Lastly, potential targets are identified that may improve photodynamic therapy against melanoma cells.

## Introduction

Melanoma is a highly aggressive malignant tumor that originates from melanocytes, and its progression is difficult to predict. Treatment of melanoma continues to face serious challenges, resulting in an increased annual global incidence of 3% (1). Melanoma typically occurs in the skin, but can also develop in other tissues that originate from pigmented neural crest (NC) cells, including the eyes, nasal cavity, anal canal, digestive tract, and genitourinary tract (2, 3). Although melanoma accounts for only 2% of all skin cancer cases, it is responsible for 80% of deaths from dermatologic cancers (4). Hence, the recent increase in morbidity and mortality due to melanoma is a matter of concern for global human health.

Current guideline-based therapies for patients with melanoma include surgery, radiotherapy, chemotherapy, immunotherapy, and targeted therapy (5). Although the treatment of patients with early stage melanoma is effective, the 5-year survival rate for advanced melanoma is only 16%, which is related to the low sensitivity to conventional treatment procedures (6). Photodynamic therapy (PDT) has been successfully used to treat patients with non-melanoma skin cancer (7), esophageal cancer (8), head and neck cancer (9), breast cancer (10), and lung cancer (11). In recent years, several *in vitro* and *in vivo* studies have been conducted to examine the efficacy of PDT for melanoma treatment; the results for which indicate that PDT may prove to be a promising adjuvant treatment for melanoma patients.

**Figure 1:**
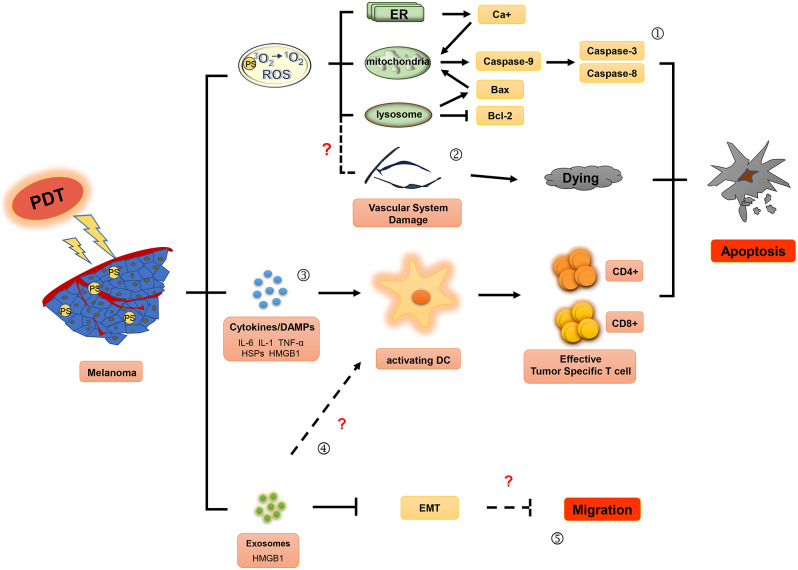
Effector mechanisms during photodynamic therapy of melanoma. The ground state photosensitizer (PS) is activated by irradiation with appropriate wavelength light to produce singlet state. Reactive oxygen species (ROS), the main cytotoxic components, can cause death of tumor cells by apoptosis (①) and induce the damage of the tumor vascular system (②). In addition, photodynamic therapy may also activate immune responses against tumors by affecting the secretion of inflammatory factor (IL-6, IL-1, and TNF-α), HSPs (heat shock proteins) and DAMPs (damage associated molecular patterns) (③), and exosomes (④). Moreover, exosomes induced by photodynamic therapy (PDT) might play an important role in inhibitory regulation of EMT (epithelial-mesenchymal transition) in melanoma cells (⑤).

**Figure 2:**
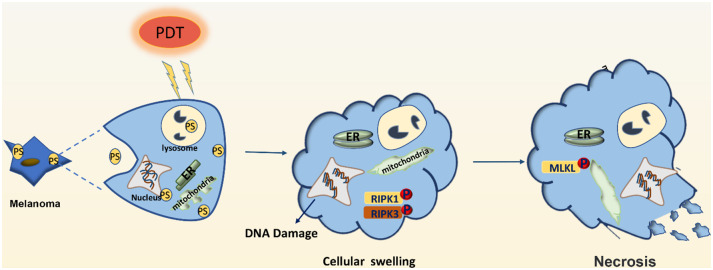
Effector mechanisms leading to necrosis after photodynamic therapy of melanoma. PDT may induce DNA damage and swelling of organelles, leading to necrosis of melanoma cells. PDT may also activate the RIPK1 pathway to promote the phosphorylation of downstream RIPK3, make the phosphorylation of RIPK3 merge with MLKL, and form RIPK1-RIPK3-MLKL complex, namely necrotizing corpuscles.

**Figure 3:**
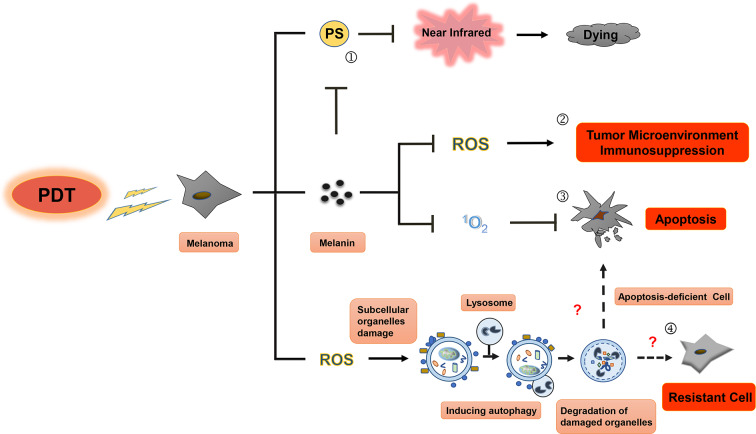
Resistance mechanisms during photodynamic therapy of melanoma. Photosensitizers cannot be effectively excited by near-infrared (NIR) in PDT for melanoma, melanin granules and autophagy could be the main contributors to this resistance. First, visible light can be absorbed by melanin in melanoma cell (①), leading to diminishment of photothermal effect induced by PS and decrease in production of ROS and singlet oxygen, then resulting in the inhibition of immune response in tumor microenvironment (②) and apoptosis blocking (③) of melanoma cell. Only in the near-infrared conditions, PS can play an even greater role in PDT treatment of melanoma. Second, subcellular organelle damage induced by ROS in PDT treatment can enhance autophagy to maintain cell homeostasis against apoptosis, which ultimately leads to the resistance to PDT treatment in melanoma (④).

Although PDT has been successfully used in the treatment of cancer and non-neoplastic diseases, its use in the treatment of patients with melanoma has been limited owing to low response rates and unsatisfactory efficiency (12, 13). This article reviews the studies on PDT treatment of melanoma and other tumors and summarizes the effects (Figures 1, 2) as well as the potential mechanisms for tolerance (Figure 3) of PDT for the treatment of melanoma patients.

### PDT

PDT is a novel non-invasive therapeutic technique for malignant tumors. The clinical results of PDT for cancer treatment show that it is efficacious in the treatment of early stage cancer that of head and neck tumors and basal cell carcinomas, for which complete remission may be achieved, which subsequently prolongs the survival time of patients with inoperable carcinoma (14, 15). The use of photosensitizers (PSs) can selectively target diseased tissues and improve the efficiency of photoinitiation. These PSs are activated by specific wavelength lasers and can trigger photochemical reactions that precisely target the tumor while reducing damage to the surrounding normal tissue. Therefore, PDT is considered to induce minimal toxicity to normal tissues and negligible systemic side effects, while significantly reducing long-term morbidity, offering positive cosmetic/esthetic outcomes, and protecting organ function (16, 17).

PDT combines photosensitizers, oxygen molecules, and light stimulation to treat tumors. Excited state singlet oxygen (^1^O_2_) serves as the primary cytotoxic material in PDT. Molecular oxygen in this state functions as a highly active reactive oxygen species (ROS) that oxidizes biological substrates (18, 19). The singlet oxygen or ROS produced within the cell membrane can cause photo-oxidative damage to proteins and lipids within the photosensitive binding site, and induce oxidative damage in the target cells, ultimately causing apoptosis, necrosis, and tumor vasculature damage. Furthermore, ROS can induce an inflammatory response to stimulate antitumor immune responses. These mechanisms, summarized in Figure 1, may lead to long-term tumor control through antitumor effects on primary/metastatic tumors (20, 21).

## PDT Mechanisms of Action

### Apoptosis and Necrosis

PDT plays an important role in cellular necrosis or apoptosis induced by light damage. The treatment method varies according to the PS aggregation site. Apoptosis is a form of programmed cell death, and apoptosis-induced tumor cell death has become one of the primary targets in tumor therapy. Alternatively, dysregulated cellular apoptosis may result in uncontrolled proliferation of melanoma cells (22). During treatment, the accumulation of PSs in the mitochondria and endoplasmic reticulum can cause oxidative stress-induced apoptosis (23). Therefore, PDT can induce apoptosis in melanoma cells, which might play a key role in inhibiting their proliferation and metastasis.

Studies have confirmed that PDT promotes apoptosis in the melanoma cell lines, A375 and UCT mel-1, by both the intrinsic and extrinsic apoptotic pathways (24, 25). PDT-induced apoptosis in melanoma cells occurs via three key signaling pathways. (1) In the intrinsic pathway, cellular death occurs through mitochondria-mediated signaling pathways involving DNA damage, p53 activation, p53-mediated inhibition of antiapoptotic proteins such as Bcl-2 (26–28), and kinases involved in cell proliferation and survival, such as B-Raf (29, 30). PSs can also induce damage in the mitochondria and lysosomes (31) by inhibiting production of MMP-9, Bcl-2, and Bcl-xL, while upregulating the expression of apoptotic related proteins Bax and PARP, and promoting activation of death receptor pathways. Moreover, PDT has been shown to induce intrinsic apoptosis by inducing increased levels of ER stress and activation of caspase cascade pathways (32). When PDT doses are increased beyond sublethal damage, stress response pathways become activated, including the ER stress pathway, with disruption of Ca^2+^ homeostasis and unfolding of protein. These responses lead to either apoptosis or autophagic cell death, dependent on the availability of Bax/Bak (33); (2) The extrinsic pathway is initiated by oligomeric death ligands, including tumor necrosis factor (TNF)-related apoptosis-inducing ligand (TRAIL) or CD95L (34); (3) Cytotoxic T lymphocytes (CTL) and natural killer (NK) cells release granzyme B, a serine protease, into the target cell to induce apoptosis (35). These cellular apoptosis pathways converge at a common terminal stage and rely on the disintegration of the cellular matrix by cysteine protease caspases. Specifically, caspase 9 is involved in the intrinsic pathway and caspase 8 in the extrinsic, both of which ultimately lead to activation of downstream caspase 3 in the execution pathway (36–38). Caspase−3,−8,−9 are released in PDT-treated melanoma cells (24, 25, 39), resulting in initiation of a series of cascades that induce irreversible apoptosis (40, 41).

PDT can also induce cellular necrosis upon accumulation of PSs in the cell membrane and lysosomes (see Figure 2). Necrosis is a caspase-8-independent cell death pathway that requires synergistic activation of receptor-interacting protein 1 (RIP1) and receptor-interacting protein 3 (RIP3) kinases (42). Unlike protein-driven cell apoptosis, necrotic apoptosis is mediated by a cascade of kinase signaling that activates (automatic) phosphorylation of RIPK1, RIPK3, and MLKL, which then form solubilized pores in the plasma membrane, leading to rapid plasma membrane rupture and inflammatory responses through the release of damage-associated molecular patterns and cytokines (43, 44). The necrosis pathway has been shown to be related to a variety of tumor types, including melanoma, pancreatic adenocarcinoma, and certain hematological malignancies (45). Mohammadalipour et al. demonstrated that a low dose of nitrogen-doped titanium dioxide (N-TiO_2_) nanoparticles (NPs) (1–100 μg/ml) stimulated autophagy flow response of non-toxic A375 cells. However, their light-activation can impede autophagosome-lysosome fusion and resulting in an increase at the basal ROS level. Therefore, PDT with N-TiO_2_ NPs leads to the blockade of autophagy flux and ultimately the occurrence of necroptosis in melanoma A375 cells (46). Additional factors that contribute to PDT-induced cellular necrosis include PS localization, exposure to light, and hypoxia-induced glucose deprivation. Furthermore, Thibaut et al. (47) demonstrated that PDT can induce necrosis in a murine melanoma B16-A45 cells signal pathway that involves activated caspase. Moreover, apoptosis and necrosis often use the same initiation signal pathway that involves activated caspase. Therefore, these are the major pathways that lead to melanoma cell death. Learning how to decrease apoptotic resistance of melanoma cells to improve PDT efficacy could be an important research goal for the future.

### Tumor Vasculature Damage

Tumor vasculature provides oxygen and nutrition to support the continuous growth of tumors and is a major pathway for metastasis (48). Therefore, antiangiogenesis treatment strategies are expected to have a strong therapeutic effect on various tumors. Antiangiogenic PDT can induce endothelial cell injury, vasoconstriction, release of coagulation factors, platelet aggregation, vascular rupture, vascular occlusion, blood flow stagnation, and hemorrhaging (49).

Lisnjak et al. (50) have demonstrated that PDT can significantly reduce the serum concentration of vascular endothelial growth factor (VEGF) as well as the metastatic transmission rate, while inducing changes to the vasculature of tumor tissues in lung carcinoma-bearing C57BL/6 mice. These results suggest that inhibition of tumor vasculature formation is an antitumor effect of PDT. In particular, the increased proliferation of vascular endothelial cells in the process of intravascular angiogenesis may lead to excessive accumulation of protoporphyrin-IX (PpIX), a potent photosensitizer, and selective enhancement of the photodynamic action on angiogenic endothelial cells in tumor tissues (51, 52). Schuitmaker et al. investigated the efficiency of the new PSs bacteriochlorin a (BCA) mediated PDT on Greene hamster melanoma implanted in the anterior eye. Following BCA-PDT, blood vessels and intracellular spaces were enlarged and clotting was immediately observed with swollen erythrocytes. Fused inner and outer membranes of mitochondria resulted in mitochondria damage that was confirmed by electron microscopy. With the passage of time, the degree of tissue and cell damage increased. At 24 h, nearly complete necrosis was observed at the treatment site. It was postulated that direct mitochondrial and vascular injury induced by BCA-PDT may account for the immediate cause of tumor necrosis (53). Similarly, Zilberstein et al. tested the effects of bacteriochlorophyll-serine (Bchl-Ser)-PDT in Nude CD1 mice bearing malignant M2R melanotic melanoma xenografts (76–212 mm^3^). Primary vascular damage with occlusive thrombi, hemorrhage, and tumor necrosis were confirmed by histopathology. Moreover, the treatment protocol was short and effective with a cure rate of over 80% (54).

Furthermore, studies have demonstrated that PDT induced vascular damage in other tumors, the mechanisms for which may translate to similar effects in melanoma. For example, ALA-PDT in a coculture of human umbilical vein endothelial cells (HUVECs) and human bladder carcinoma cell line had an antiangiogenic and antitumor effect, most notably when in combination with deferoxamine, which increased accumulation of PpIX (55). Karwicka demonstrated that treatment focused on vascular destruction (V-PDT) can lead to highly effective long-term antitumor responses mediated by strong blood supply deprivation *in vivo*. Further, 67% of Lewis lung carcinoma (LLC) bearing mice treated with V-PDT exhibited complete regression without relapse for over 1 year (56). Similarly, an *in vivo* study performed by Inoue et al. (55) showed that antiangiogenic PDT is effective for tumor tissues and does not significantly affect angiogenesis in normal tissue surrounding tumors in lung carcinoma-bearing C57BL/6 mice.

Although there is limited direct evidence demonstrating that PDT damages blood vessels in melanoma tumors, specifically, the results of the aforementioned studies do offer a theoretical basis for this hypothesis. Antiangiogenic PDT may function to disrupt or damage the tumor vasculature of melanoma; therefore, the combination of antiangiogenic PDT with radiochemotherapy may be clinically effective in relieving symptoms and improving the survival rate of patients with melanoma. The optimal approach to tumor and vascular targeting of PDT can disrupt melanoma and endothelial tumor cells and activate the immune response, thereby improving overall efficacy (16, 57).

### Antitumor Immune Response

Immune tolerance in the tumor microenvironment reduces the tumor killing capacity of immune cells and promotes tumor cell growth (58). Hence, reversion of immunosuppression in the tumor microenvironment is currently an exciting area in tumor immunotherapy research. Preclinical studies have demonstrated that PDT enhances host antitumor immune responses in lung cancer and non-melanoma skin cancer, at the treatment site by inducing oxidative stress, which can trigger the release of proinflammatory factors such as TNF, interleukin (IL)-6, IL-1, heat shock proteins (HSPs), complement proteins, and metabolites (17, 59). Innate immune cells including monocytes/macrophages, neutrophils, and dendritic cells may then be recruited to the treatment site by these inflammatory cytokines, which then function to kill tumor cells. Tumor vasculature also changes significantly upon PDT-induced inflammation. Adhesion molecules (intracellular adhesion molecules-1, vascular cell adhesion molecules-1, and selectins), which were found to be overexpressed following PDT, can recruit neutrophils and other inflammatory cells to tumor sites and convert the tumor vascular endothelium from a non-thrombotic, non-adhesive barrier between blood and tumor tissue to a pro-adhesive surface permitting infiltration of blood constituents (60). As a result of increased permeability, inflammatory cells have been shown to readily enter the vasculature after which the innate immune cells infiltrate the subcutaneous FsaR fibrosarcoma tumors in syngeneic C3H/HeN mice (61).

Acute inflammatory responses are associated with the development of adaptive antitumor immunity and thus, can protect the host organism in an antigen-specific manner. Previous studies have confirmed that PDT primarily activates dendritic cells (DCs) to enhance adaptive antitumor immunity (62, 63); and damage-associated molecular-pattern molecules (DAMPs)/cell death-associated molecular-pattern molecules (CDAMPs) that become released from dying tumor cells may be involved in this process. HSP70, a key member of the HSP complex, is released after PDT and binds to tumor cytoplasmic antigens in a stable concomitant complex (64). Thereafter, HSP-tumor antigen complexes bind to risk signal receptors and are recognized by Toll-like receptors 2 and 4 on the surface of the DCs (65). In turn, these induce activation of DCs and the release of proinflammatory cytokines.

Previous studies have demonstrated that the expression and secretion of high mobility group box 1 (HMGB1) in mouse colon cancer cells (66), cutaneous squamous cell carcinoma (SCC) cells (67), LLC cells (68), and cervical cancer cells (69) are significantly elevated following PDT. Extracellular HMGB1 can activate macrophages and DCs, and recruit neutrophils, using various receptors. Korbelik et al. (68) suggested that PDT-treated LLC cells release signals to induce production of HMGB1 by macrophages and other immune cells. These signals may then promote an antitumor immune response. Furthermore, a clinical study has suggested that the percentages of mature DCs increases in the blood of patients treated with 5-aminolevulinic acid-mediated PDT (ALA-PDT). Moreover, ALA-PDT significantly downregulated miR-34a and upregulated HMGB1 expression levels in cervical cancer tissues (69). PDT was also reported to induce a further increase in the number of regulatory T cells and NK cells and upregulate HMGB1 expression in the peripheral blood of patients with head and neck squamous cell carcinoma (HNSCC) (70). Cumulatively, these results suggest that PDT induces HMGB1 expression and is a crucial pathway for activating antitumor immunity.

Several proinflammation cytokines and DAMPS induced by PDT in the tumor microenvironment play a critical role in activating DCs. Mature DCs migrate to lymph nodes in large numbers and upregulate the expression of major histocompatibility complex (MHC)-I, MHC-II, and costimulatory molecules CD80 and CD86 (71). These changes enable DCs to express the antigen peptide-MHC complex on their cell surface and enhance activation of CD4+ T helper cells and CD8+ CTLs, thus triggering an adaptive immune response against tumor antigens (72).

Recent studies have shown that immune checkpoint inhibitors against programmed death 1 (PD-1) and programmed death ligand 1 (PD-L1) are well-established leading immunomodulatory agents that act in specific pathways involved in the adaptive immune suppression of tumor tissues. The focus of these studies was initially placed on targeting cancers that were considered to be immunogenic, including melanoma, renal, and lung cancers; however, subsequently the application was expanded to include other cancers such as Hodgkin lymphoma, urothelial, as well as head and neck cancer (73). Wang et al. (74) demonstrated a multifunctional acid-activated micro-micelle that enhances a PDT-driven tumor immune response by inhibiting the expression of PD-L1 in melanoma cells. This micelle not only enhances ROS-induced and PD-L1 knockout antitumor immune characteristics but also stimulates the immune response by promoting cytokine secretion and lymphocyte proliferation, and effectively inhibits B16-F10 melanoma tumor growth. Due to the immune response and immunologic memory induced by PDT, pulmonary metastasis of transplanted B16-F10 melanoma xenograft tumors was also inhibited in the *in vivo* study. These studies have comprehensively demonstrated that PDT-induced antitumor immunity plays an important role in the treatment of melanoma.

Additional studies have shown that PDT combined immunotherapy induces potent systemic antitumor immunity in mice and should be evaluated for the treatment of human cancer. Saji et al. showed that although PDT and (intratumorally injection of naïve dendritic cells) IT-DC were not effective on their own, PDT combined with IT-DC eradicated both CT26 and B16 tumors in a significant proportion of animals, and prolonged the survival of mice with tumors that were not cured. Most importantly, PDT combined with IT-DC treatment at a single tumor site resulted in tumor regression at distant sites, including multiple lung metastases (75).

*In situ* photoimmunotherapy (ISPI), which combines photodynamic therapy with immunological stimulation induction, is a promising modality for the treatment of metastatic melanoma (76). A continued local application of topical imiquimod (a Toll-like receptor 7 agonist) in combination with indocyanine green-PDT has been used to treat late stage melanoma patients. In one study, 11 patients received ISPI in one or multiple 6-week treatment cycles applied to a 200-cm^2^ site, which often contained multiple cutaneous metastases. The treatment included local application of topical imiquimod, injection of indocyanine green (ICG), and a 805 nm laser for local irradiation. All patients completed at least one treatment cycle. The result shows that complete response was observed in six patients, five patients were alive at the time of last follow-up and the probability of 12-month overall survival was 70% (77). Therefore, ISPI not only produces a complete local response but also demonstrates an effective immune response against metastatic nodules. Furthermore, injecting dendritic cells (DCs) into a tumor can stimulate an immune response, which, combined with local PDT, induces a striking antitumor effect with potent systemic antitumor immunity. In fact, PDT + IT-DC eradicated both CT26 and B16 tumors in a significant proportion of animals and prolonged the survival of mice in which the tumors were not cured (75). PDT creates a favorable microenvironment for the acquisition of tumor antigen and the activation of DC, which reduces the need for tumor antigen loading *in vitro* by DCs (78). These studies show that these combined treatments can sometimes induce strong and durable tumor specific immunity that results in destruction of targeted tumors as well as initiation of systemic antitumor immune response.

Therefore, PDT combined with immunostimulatory agents seems to show great promise and could change the current therapeutic strategy for melanoma treatment. However, to improve its efficacy, further investigations on the precise antitumor immune mechanism elicited by PDT in melanoma treatment must be completed.

### Exosomes

Exosomes are a type of extracellular particle that can mediate the communication between cells. They contain cellular components such as microRNAs, mRNAs, proteins, and DNA. Exosomes have been a subject of recent investigation to study the mechanisms of tumorigenesis and tumor progression, including tumor metastasis, angiogenesis, antitumor immunity, and tumor immunological escape (79–81). Two studies have demonstrated that PDT can regulate exosome secretion by tumor cells. ALA-PDT induced the production of exosomes with high levels of HMGB1, which in turn promoted DC maturation in the peripheral blood of ALA-PDT-treated patients with cervical cancer (69). Moreover, exosomes induced by PDT treatment were involved in the regulation of epithelial-mesenchymal transition (EMT) of tumor cells. Analysis of the exosomes obtained from the plasma of nine HNSCC patients (three in stage pT1, one in stage pT3, and five in stage pT4), all of whom benefited from positive clinical outcomes following treatment, on day 7 or 4–6 weeks after PDT treatment confirmed that the PDT-mediated secretion of exosomes contained E-cadherin, and restored epithelial morphology and epithelial cell adhesion molecule (EpCAM) expression in tumor cells. Further, the exosomes of these patients exhibited downregulated expression of mesenchymal genes and inhibited proliferation, migration, and invasion of the recipient tumor cells *in vitro* (82). These studies suggest that PDT can potentially induce antitumor immune responses and inhibit tumor migration by regulating exosome secretion. However, the key components of exosomes involved in PDT-mediated tumor suppression are currently unknown, which may be a hot topic of future studies.

## Resistance Mechanisms for PDT

### Melanin Pigment

Melanin can absorb ultraviolet (UV) and visible light, which can prevent ultraviolet radiation (UVR) to protect the skin (83). The melanin pigment is synthesized in melanocytes by tyrosinase that is integrated into an organelle named melanosome (84, 85). After synthesis, melanin is transported into the surrounding keratinocytes of the epidermis (86).

Tyrosinase (TYR) is a key enzyme that produces melanin from melanocytes. Tyrosinase-related proteins (TRP-1, TRP-2) convert tyrosine to dopamine and dopamine to dopamine quinone in a two-step enzymatic reaction catalyzed by tyrosine hydroxylase and dopamine oxidase, respectively. The resulting quinone is used to synthesize pheomelanin and eumelanin (87). Melanogenesis can be induced by a variety of paracrine cytokines, including α-melanocyte-stimulating hormone (α-MSH), endothelin 1 (ET-1), nitric oxide, adreno-cortico-tropic-hormone (ACTH), prostaglandins, thymidine dinucleotides, and histamines, upon exposure to ultraviolet B (UVB) (88). These factors activate pigment-related proteins such as microphthalmia-associated transcription factor (MITF), tyrosinase (TYR), tyrosinase-related protein-1 (TRP-1), and tyrosinase-related protein-2 (TRP-2) (89). MITF, in particular, plays a key role in melanogenesis by regulating melanocyte differentiation, pigmentation, proliferation, and survival. In the absence of radiation, melanocytes are exposed to eumelanin that causes DNA damage by inducing DNA strand breakage. In addition, melanin may damage DNA through a Fenton reaction (90), and can prevent the access of DNA repair enzymes to the DNA damaged sites. Furthermore, DNA damage can cause melanocytic mutation and increase melanin production (91). Moderate to high levels of pigmentation have been observed in melanoma tumors, and the melanin content in cells may be directly proportional to the degree of cell differentiation and inversely proportional to cell growth (92).

The singlet oxygen produced by PDT can reduce natural oxidation of melanin and DNA damage caused by melanin (93). PDT has been suggested to reduce the melanin content and tyrosinase activity in melanocytes, but not to affect cell survival (94). Studies have shown that melanin can scavenge ROS, such as singlet oxygen, hydroxyl radicals, and superoxide anions (95). These studies indicate that melanin protects pigmented cells from oxidative stress, changes cell metabolism, induces immune suppression and mutagenesis of tumor microenvironment, thus protecting malignant melanocytes from various treatments. Currently, it is believed that the inhibition of melanogenesis by immunotherapy, radiotherapy, chemotherapy, and PDT can reduce the incidence of melanoma deterioration (96).

In order to avoid an adverse reduction in PDT efficacy due to the absorption of light by melanin, combination with depigmentation may be necessary. *In vivo* studies demonstrated that combining hypericin-mediated PDT with depigmentation agents, such as tyrosinase inhibitors (kojic acid) or phenyl thiourea, significantly increases ROS production and decreases viability of MEL-1 cells to a similar extent as that of A375 cells, suggesting that this treatment increases susceptibility of melanoma cells (97, 98). Further, melanoma cells were treated by photobleaching in combination with PDT and 420-nm violet light (99), and the results showed that the bleaching effect of violet light on melanoma cells significantly increased their sensitivity to PDT. Therefore, a drug with the ability to inhibit melanin production or induce depigmentation would be an important component in the therapeutic arsenal to treat melanoma more effectively.

### Autophagy

Autophagy is a basic physiological process that relies on lysosomal pathways to degrade cytoplasmic proteins and organelles to maintain cellular homeostasis. Interestingly, autophagy elicits opposing effects depending on the needs of the cells. For instance, it can serve as a type II programmed cell death pathway if necessary, while eliciting cytoprotective effects in other instances. In the advanced stage of melanoma and many other types of tumors, autophagy serves as a resistance mechanism and occurs as a tumor cell pro-survival mechanism (100, 101). Several studies have shown that chemotherapy, radiotherapy, immunotherapy, and PDT can upregulate the expression of autophagy-related proteins such as ATG4, ATG5, ATG12, and Beclin-1, thereby increasing tumor resistance (102–106). Marino et al. demonstrated that melanoma cells can survive in an acidic environment by upregulating autophagy; meanwhile, inhibition of ATG5 can reduce survival of melanoma cells (107). Similarly, Mehnert et al. established a mouse melanoma model by deleting ATG7 and PTEN gene in melanoma cells (108), which significantly inhibited tumor growth and prolonged the survival of mice. In this model, dabrafenib combined with ATG7 antagonistic therapy significantly inhibited the growth of melanoma (109). Martin et al. (110) also demonstrated that the combination of chemotherapy with autophagy and mitogen extracellular signal-regulated kinase (MEK) inhibition can enhance the melanoma cell killing effect of chemotherapeutic drugs. Based on these studies, the induction of autophagy may serve as a resistance mechanism to PDT for melanoma treatment.

After PDT treatment of tumor cells, autophagy is activated through the cell-related pressure sensor, and the intracellular components or organelles are transported to lysosomes for decomposition and reuse to offset the alarms of their environment and to respond to the cytotoxic effect. Tumor cells need to control and adapt to the redox imbalance caused by ROS produced following PDT. The redox homeostasis is closely related to the occurrence, progression, and metastasis of tumors (111). PDT-induced autophagy provides a protective mechanism for breast cancer cells, osteosarcoma cells, HeLa cells, and colon and rectum cancer stem cells. Inhibition of autophagy can enhance the photodynamic tumor cell killing effect (112–114). On the other hand, PDT-induced autophagy can also lead to the dissociation of Bcl-2 and Beclin-1, reduce Bax/Bak protein levels, and inhibit caspase-8 through induction of apoptosis. However, these results suggest that autophagy-induced cell death can only occur in tumor cells with defective apoptosis (115).

In addition to directly destroying cancer cells, PDT seems to influence other indirect killing mechanisms by, for instance, regulating innate antitumor immune activity and damaging tumor vasculature (116). Autophagy is fundamental for cell survival and for the proper function of immune cells and endothelial cells; however, its role in determining melanoma resistance to PDT is still unknown (117–119). Therefore, further studies on understanding the molecular mechanism of PDT-induced autophagy in melanoma resistance will be of significance to improve the development of future combinatorial strategies.

### Photosensitizers

PSs accumulated intracellularly are the first step in PDT. Given the role of singlet oxygen and other ROS in tissue damage inflicted by PDT, the most commonly used PSs are dependent on the molecular oxygen tissue concentrations. Using PSs in PDT allows for selective tumor targeting due to the intracellular metabolism, and are, therefore, important for effective PDT treatment of malignant tumors. However, melanoma patients do not seem to have benefited significantly from PDT using the PSs and treatment protocols available today. There are two possible reasons that might account for this. First, the high melanin content in melanoma cells absorbs visible spectral radiation, especially in the blue region, which reduces the light available for absorption by the PS and reduces PDT efficacy (120). PSs with absorption in the near-infrared (NIR) range may be more suitable for PDT of melanoma cells. Second, tumor targeting and PS accumulation may not be fully effective with current PSs. Therefore, in this article, we will now identify and describe PSs potentially available for melanoma treatment.

Generally, PSs for PDT are divided into three generations, some of which have been investigated for melanoma treatment (Table 1). Porfimer sodium, the first-generation PS, mediated PDT on melanotic and amelanotic malignant melanoma in athymic nude mice and was effective against amelanotic but not melanotic melanoma (121–124). There are many types of second generation PSs, such as porphyrin derivatives (PD), phthalocyanines, biomimetic dyes, and polycyclic quinone. 5-ALA is one of the PD used for non-melanoma clinical treatment. Many *in vitro* studies revealed that 5-ALA-PDT effectively inhibits the activity of melanoma cell lines and triggers cellular apoptosis (24, 31, 125–129). However, when 5-ALA was used for cutaneous melanoma treatment *in vivo*, it did not significantly inhibit tumor progression (24). The effect of 5-ALA seems to be related to the dose. Our unpublished data showed that only high doses of 5-ALA (10 mM) significantly inhibited autophagy in melanoma cells. As reviewed in Table 1, methylene blue (MB) is a cationic dye derived from the phenothiazine family. It exhibits strong broad-spectrum red light absorption (550–700 nm, maximum absorption at 664 nm) and high affinity for melanocytes, which helps selective absorbance of this PS in cutaneous melanoma. MB-PDT and irradiation with a 664-nm light hindered tumor growth and prolonged survival in a B16F10 pigmented mouse melanoma model (163, 164).

**Table 1:** The studies of PSs used during PDT to treat melanoma.

**Classification**		**PS**	**Cell/Tumor**	**Wavelength**	**References**	
First generation	Hematoporphyrin Derivative (HpD)	Porfimer sodium	MMCs MMC tumor bearing mice (in vivo^*^); YUSAC2/T34A-C4; Human Beidegröm Melanoma cell line	Dye-laser (630 nm); Red light (570–650 nm)	(121–124)	
Second generation	Porphyrin derivatives (PD)	5-ALA	Mel25; G361; A375; WM451Lu; B16; SKMel-23; SKMel-28	Red light (635 nm);Halogen lamp (420–1,400 nm)	(24, 31, 125–129)	
		Ruthenium porphyrins	ME300	Red light (652 nm)	(130)	
		Halogenated porphyrins	A375	Red light (633 nm)	(131)	
		Verteporfin	YUSAC2; T34A-C4	Visible light	(121)	
		mTHPC	B16		(47)	
	Phthalocyanines	Zinc octacarboxyphthalocyanine(ZnPcOC)	Me45	Diode laser (685 nm)	(132)	
		Aluminums(III) phthalocyanine chloride tetrasulfonate (AlPcSCl)	A375	Red light (682 nm)	(133)	
		Dichlorosilicon phthalocyanine (Cl2SiPc)	M6	Red light (683 nm)	(134)	
		Chloroaluminum Pc (ClAlPc)	M3Dau	Red light (670 nm)	(135)	
		Ruthenium porphyrins	M6	Red light (652 nm)	(130)	
		Chloroaluminum phthalocyanine (ClAlPc)	G361 B19	Diode laser (670 nm)	(136, 137)	
		ClAlPcS (2)	G361	Red light (635 nm)	(127)	
		Chlorin e 6(Ce6)	B16; B16 tumor bearing mice (in vivo^*^)	Red light (664 nm)	(127, 138–141)	
	Biomimetic dye	Methylene blue (MB)	B16F1; B16F1 tumor bearing (in vivo^*^); C57BL/6J mice; SK-23; SK-Mel 28	Diode laser (650 nm)	(142, 143)	
	Polycyclic quinone	Hypericin	UCT Mel-1; A375; UCT Mel-3	UVA (400–315 nm) or 594 nm	(133, 144, 145)	
		ZnTPPS(4)	G361	Red light (635 nm)	(127)	
Third generation	Cross linked with the second generation	CDG2/5-ALA/HA	B16; A375	Red light (635 nm)	(146)	
		5-ALA/DPPC5-ALA in 1,2- dipalmitoyl-sn-glycero-3-phosphocholine	B16-F10	Red light (630 nm)	(147)	
		5-ALA-silver nanoparticles	B16-F10	Red light (635 nm)	(148)	
		Ver-MSNs	B16-F10; B16 tumor bearing mice (in vivo^*^)	Red light (693 nm)	(149)	
		ZnTcPc and ZnTcPc-g attached to gold nanorods	B16-F10; B16-G4F	Red light (635 nm)	(150)	
		liposomes-encapsulated Fe-CHL.	B16-F10	Red light (652 nm)	(120)	
		ClAlPc-loaded SLNs	B16-F10	Diode laser (670 nm)	(151)	
	Nanometer materials	nitrogen-doped Titanium dioxide	B16-F10	Ultraviolet light	(152)	
		RB-MMSNs	B16; B16 tumor bearing C57BL/6J mice (in vivo^*^)	NIB	(153)	
		TAG	B16F1 tumor bearing mice (in vivo^*^)	Simulated sunlight	(154)	
		DOX/PheoA-ALG NPs	B16; B16 tumor bearing C57BL/6J mice (in vivo^*^)	Red light (670 nm)	(155)	
		POP micelles	B16-F10 tumor model (in vivo^*^)	Red light (671 nm)	(74)	
	NIR absorbing	5-Benzoindotricarbocyanine (indocyanine green, ICG)	Sk-Mel-28; S91	Pulsed, 788 nm	(156, 157)	
		PNPG-PEG-HA	B16; B16 tumor bearing mice (in vivo^*^)	Red light (808 nm)	(158)	
		Tookad	M2R; Tumor C57BL/6 mice bearing (in vivo^*^)	Diode laser (763 nm)	(159)	
		Naphthalocyanines	B78H1 B78H1 tumor bearing C57BL/6J mice (in vivo^*^)	Ti: sapphire laser 809 nm (Pt) or 826 nm (Pd)	(160)	
		PcNP@Drug	SKMEL-28; SKMEL-28 tumor bearing mice (in vivo^*^)	Red light (730 nm)	(161)	
		Platinum(II) Ring-Fused Chlorin	A375	Red light (700–850 nm)	(162)	

* *In vivo indicated animal experiment*.

*Ver-MSNs, verteporfin-loaded mesoporous silica nanoparticles; RB-MMSNs, polyethylene glycol–polyaspartate-modified rose bengal-loaded magnetic mesoporous silica; TAG, titanium-dioxide-nanoparticle-gold-nanocluster-graphene; DOX/PheoA-ALG NPs, anticancer agent, doxorubicin (DOX), was also loaded within the PheoA-ALG nanoparticles (DOX/PheoA-ALG NPs); PNPG-PEG-HA, Poly(N-phenylglycine)-based nanoparticles*.

The third generation PSs have demonstrated improved tumor treatment characteristics, such as higher biocompatibility, stronger tumor targeting capacity, higher ROS generation rate, and longer wavelength absorption, compared to the first- and second generation PSs. This was achieved by incorporating the PS into a nanocarrier and by using peptides or antibodies for selective delivery of PSs to tumors. The second generation PSs combined with carrier can circumvent the skin barrier and improve selective delivery to melanoma cells (120, 146, 149, 150, 165). The use of nanocarriers can improve the activity of photosensitizing agents through preferential accumulation of the carrier at the tumor site and facilitating slow, controlled release of the PS (166). Nanocarriers function by binding to the target molecules of overexpressed receptors in tumor cells, leading to enhanced uptake of PS that have been conjugated with peptides, aptamers, and antibody fragments. Additionally, PS-coated upconversion nanoparticles were found to trigger ROS production under 980 nm NIR excitation and showed great promise for PDT (167). Rationally designed DNA nanosponges were reported to load and deliver PSs effectively, target tumors precisely, and effectively relieve hypoxia-associated resistance to enhance the efficacy of PDT (168). In general, the application of nanotechnology in PDT aims to improve water hydrophobic drug compatibility/PS, protect against drug degradation, produce a sustained release of drugs, improve drug bioavailability (169), increase tumor selectivity, and allow improved treatment of deep tumor infiltration depth, so as to increase therapeutic efficacy and reduce adverse side effects (170–172).

It has been found that micelles, liposomes, and metal oxide enable passive targeting of tumors through enhanced permeability and retention (EPR) effects to improve the efficacy of PDT (173, 174). Many of these PS delivery systems have been used in melanoma treatment (175, 176). For example, nitrogen-doped titanium dioxide, polyethylene glycol-polyaspartate-modified rose bengal-loaded magnetic mesoporous silica (RB-MMSNs), Titanium-dioxide-nanoparticle-gold-nanocluster-graphene (TAG), doxorubicin, which is an anticancer agent (DOX/PheoA-ALG NPs), and POP micelles have all been shown to increase PDT efficacy against B16F10 melanoma *in vivo* and to stimulate and enhance immunological responses (152–155).

NIR light absorption PSs also improve the photodynamic effect. P-nitrophenyl-pD-glucopyranoside (PNPG), a new class of PS, exhibits large absorption peak in the NIR spectrum. The encouraging results have revealed that PNPG can effectively target CD44-overexpressing cancer cells and selectively kill B16 cells when exposed to NIR light (808 nm) after modified with hyaluronic acid (HA) and polyethylene glycol diamine (PEG) (158). The ICG-mediated PDT with a broader irradiation range (600–1,600 nm) was studied previously. The authors found that NIR radiation was most effective in inducing B16F10 cell apoptosis and G0/G1 cell cycle arrest *in vitro* (156, 157). In addition, Tookad1 (177), naphthalocyanines (160), PcNP@Drug (161), and Platinum(II) Ring-Fused Chlorin (162) were reported to kill melanoma cells and suppress malignant melanoma tumor growth upon exposure to NIR light (700–1,000 nm) in a pigmented melanoma model. Therefore, third generation PSs for melanoma treatment are currently in the spotlight of the PDT research field.

## Sonodynamic Therapy and Photothermal Therapy

Branching out from in-depth research on PDT, new treatments have emerged such as sonodynamic therapy (SDT) or photothermal therapy (PTT), which have advantages similar to PDT, including tumor selectivity, minimal invasiveness, and ability to enhanced PS activation without the need for direct access to the tumor site.

SDT represents an emerging approach that offers the possibility of non-invasively eradicating solid tumors in a site directed manner. It involves the sensitization of target tissues with a non-toxic sensitizing chemical agent and subsequent exposure of the sensitized tissues to relatively low intensity ultrasound. Because ultrasound has stronger tissue penetration ability, this method has advantages over similar alternative methods (such as PDT), thus showing more concentrated therapeutic effect on the lesions. Many experiments have confirmed that SDT has obvious killing effect on tumor cells at home and abroad. Jin et al. (178) compared the efficacy of ALA-PDT and SDT in a squamous cell carcinoma (A431) cell line as well as the ability of these treatments to reduce the size of A431 ectopic tumors in mice. Similarly, the relative efficacy of Rose Bengal-PDT and SDT was investigated in a B16-melanoma cell line and in a B16 ectopic tumor model. The results tested no statistically significant difference in efficacy between ALA -PDT or SDT in the non-melanoma model; however, Rose Bengal-SDT was significantly more efficacious than PDT in the melanoma. This difference in efficacy was due to the pigmentation of the melanoma cells that effectively filtered the excitation light preventing it from activating the sensitizer, while the use of ultrasound avoids this problem (179). Harada et al. also demonstrated the induction of the melanoma cell (C32) apoptosis by the combination of TiO_2_ nanoparticles and ultrasound (US). Meanwhile, *in vivo* results showed significant inhibition of C32 solid tumors in mice growth in groups treated with TiO_2_ and US (TiO_2_-SDT) (180). ALA-SDT showed synergistic antitumor effects in malignant melanoma by constituting a positive feedback loop of p53-miR-34a-Sirt1 axis (181). In addition, there are several new sonosensitizer-mediated sonodynamic therapies that result in complete regression of melanoma, such as chloroaluminum phthalocyanine disulfonate (ClAlPcS2), a nickel ferrite/carbon nanocomposite (NiFeO/C), redox/enzyme/ultrasound responsive chondroitin sulfate-chlorin e6-lipoic acid nanoplatform loading docetaxel (136, 182, 183). These studies suggest that SDT may be more effective than PDT in treating hyperpigmented melanoma.

PTT is an emerging approach for tumor treatment. Under NIR illumination, the photothermal conversion materials can convert light energy into heat energy to kill tumor cells. The damaged tumor cells can evoke efficient antitumor immune response and promote the necrosis and apoptosis of tumor cells. PTT provides a precise and minimally invasive alternative for cancer treatment. It is effective in controlling metastatic cancer. Xu et al. (184) developed the GNS-TAT-Cy5 nanoprobe which can serve as a precise theranostic platform via regulating the photothermal dose and achieved regulation and detection of apoptosis related to caspase-3 for melanoma. Zhang et al. (185) demonstrated the potential application of a piTRL-mediated immuno-photothermal therapy against melanoma and its metastases in a study *in vivo*. Alvi et al. (186) described a convenient method to synthesize a new type of superparamagnetic up conversion nanoprobes, which possesses high biocompatibility and can be used in imaging-guided photothermal therapy for the treatment of malignant melanoma. PTT has advantages similar to PDT, such as high specificity, minimal invasiveness, and precise spatial-temporal selectivity. PTT penetrates deeper into the tissue, making it more effective in treating melanoma.

## Clinical Cases of PDT in Melanoma

Although the question of whether PDT can be used in the clinical treatment of melanoma still remains unanswered, some reports on clinical application of PDT for melanoma treatment are available. For instance, Barbazetto et al. reported results of PDT in four patients with choroidal melanoma. Results showed PDT led to tumor regression in two patients (one tumor decreased in size and remained stable for 18 months; another tumor exhibited no growth for 11 months), melanomas in the other patients continued to grow, eventually requiring surgical intervention (187). Interestingly, another study used the same protocol on a patient with choroidal amelanotic melanoma and reported complete resolution of the lesion, leaving a flat, atrophic chorioretinal scar. Thirteen months after her last treatment, she remained asymptomatic with no signs of recurrence (188).

Alternatively, Campbell et al. tested the effectiveness of PDT with verteporfin on nine patients with posteriorly located amelanotic choroidal melanomas, one of which contained a pigmented portion. The basal diameters of the tumors ranged from 4 to 16 mm and heights ranged from 1.3 to 5.7 mm. Treatment was repeated until the melanoma adopted a flat appearance or its height reached a stable end point. Combination therapy resulted in complete tumor regression with no recurrence in eight patients during a follow-up period between 34 and 81 months. However, one case in this study presented with two local recurrences, one at 21 months and the other at 34 months (189). Similarly, O'Day et al. reported that initial tumor regression was achieved in 36 of 41 (88%) patients with choroidal amelanotic melanoma (no distant metastasis) following an initial course of PDT. However, recurrent disease occurred in 44% of these cases with a mean follow-up of 3.5 years. Moreover, primary treatment failure occurred in 12% of patients (190). Turkoglu et al. also examined the effects of PDT on 12 patients with eye melanoma; 10 of whom demonstrating amelanotic and two presented with a lightly pigmented appearance. Results showed complete tumor regression of small amelanotic choroidal melanoma in 67% of patients at a mean of 5 years (191). Similarly, Fabian et al. (192) reported PDT to be a safe and efficient treatment modality for small pigmented posterior pole choroidal melanoma, achieving short-term tumor control in 80% of patients by 6-month follow-up after three PDT sessions. Cumulatively, these studies suggest that PDT may be an effective therapy strategy for choroid melanoma, with no major effect on visual acuity. Alternatively, Sheleg demonstrated the effectiveness of Ce6-PDT on skin metastases of pigmented melanoma (193). Above all, PDT may induce tumor regression in a significant proportion of melanoma.

Although PDT has proven to be relatively safe for use in clinical treatment, minor side effects have been reported, the most common of which is local swelling, pain, and a burning sensation (194, 195). PDT may also elicit side effects such as skin rash, as was reported in two patients with early gastric cancer (196). Furthermore, PDT treatment of esophageal cancer may cause mild esophageal stenosis (197). Nevertheless, an increasing number of studies suggest that PDT appears to be a non-invasive, relatively simple method that can be performed on an outpatient basis. It has also demonstrated reproducible results in basal-cell carcinoma, cervical intraepithelial neoplasia, and cervical human papilloma virus (HPV) infection cases, while causing minimal side effects (15, 198, 199).

## Future Directions

Despite the efficacy of surgical treatment for early stage melanoma, appropriate diagnosis of this condition is often difficult, which causes delay in treatment. Melanoma is often not diagnosed until intermediate or late stages, which translates to poor prognosis, recurrence, and low survival rate. Therefore, the discovery of new adjuvant treatments is an important and valuable subject in the field of melanoma research.

PDT is a promising therapeutic strategy for tumor treatment. Significant breakthroughs in basic research have indicated that PDT can provide substantial benefits in the treatment of advanced stage melanoma. Overall, this review has summarized efficacy and resistance mechanisms of melanoma during PDT treatment, and described new adjuvant therapeutic approaches. The synthesis of new PSs that absorb NIR light may improve the efficacy of PDT treatment. Moreover, PDT combined with autophagy inhibitors, immunotherapy, or melanogenesis inhibitors might be a better treatment to overcome melanoma resistance and achieve better therapeutic effects. However, for optimal safety and efficacy, it will be very important to understand the molecular mechanisms of these combination therapies. For example, tumor-derived exosomes induced by PDT might be a double-edged sword for melanoma treatment. At present, what we can definitely say is that further research on ways to exploit PDT for melanoma treatment should continue to be an important focus of future research.

## Author Contributions

Y-GL and HZ contributed substantially to the overall concept of this review, revised the article, and they gave the approval of the final version for publishing. X-YL and L-CT wrote the manuscript. L-WD and XL prepared the figures. W-QZ and X-XS prepared the table.

## Conflict of Interest

The authors declare that the research was conducted in the absence of any commercial or financial relationships that could be construed as a potential conflict of interest.
